# Decreased total antioxidants status in the plasma of patients with pseudoexfoliation glaucoma

**Published:** 2011-10-25

**Authors:** Khaled K. Abu-Amero, Altaf A. Kondkar, Ahmed Mousa, Essam A. Osman, Saleh A. Al-Obeidan

**Affiliations:** 1 Department of Ophthalmology, College of Medicine, King Saud University, Riyadh, Saudi Arabia; 2 Department of Ophthalmology, College of Medicine, University of Florida, Jacksonville, FL

## Abstract

**Purpose:**

To evaluate total antioxidant status (TAS) in the plasma of pseudoexfoliation glaucoma (PEG) patients and to compare this level with a matching control group. Additionally, we aim to investigate the effect of the combined action of the lysyl oxidase-like 1 (*LOXL1*) mutation status with TAS level on the development of PEG.

**Methods:**

Plasma samples were obtained from 54 PEG patients and 54 controls of matching age, sex, and ethnicity. TAS in all samples was determined by spectrophotometric and enzyme-linked immunosorbent assay methods. The coding region of *LOXL1*, where it encompasses both single nucleotide polymorphisms (SNPs; rs1048661 and rs3825942), was sequenced.

**Results:**

The mean (±SD) total antioxidant (TAS) value was lower among patients: 0.87 (0.24), range 0.9–1.41 than controls: 1.07 (0.23), range 0.72–1.94, and this difference was statistically significant (p<0.0001: 95%CI: −0.295–0.114). Evaluating the impact of age, sex, and the mutation in addition to the mean TAS value in patients with PEG, a logistic regression analysis was conducted using diseased/not diseased as the outcome of interest (the dependent variable). Results show that, controlling for all other variables, mean TAS value (p<0.0001) and the mutation G/G in rs3825942 (p=0.041) are significant risk factors for PEG.

**Conclusions:**

Our findings provide evidence that TAS decreases in the plasma of PEG patients, suggesting that TAS may have an important role in the pathogenesis of PEG. The combined effect of the “G” allele and the decreased TAS may contribute to the overall pathogenesis of PEG.

## Introduction

It has been demonstrated that oxidative DNA damage is significantly greater in the trabecular meshwork (TM) cells of glaucoma patients compared to controls [1]. Moreover, in vivo studies in humans have shown that both intraocular pressure (IOP) increase and visual field damage are significantly related to the amount of oxidative DNA damage [2]. Similarly, severity of optic nerve damage in eyes with primary open-angle glaucoma is correlated with changes in the TM [3]. It has been shown that the retinal ganglion cells (RGCs), which may die through an apoptotic process, lead to glaucomatous optic neuropathy [4,5]. In addition to elevated intraocular pressure (IOP) [6,7], retinal ischemia [8], nutritional status [9], and oxidative stress have been proposed as etiologic factors in the pathophysiology of glaucomatous RGC death [10]. Oxidative stress has been implicated to cause increased IOP by triggering TM degeneration and thus contributing to alterations in the aqueous outflow pathway [11]. Indeed, treatment with hydrogen peroxide (H_2_O_2_) impairs TM cell adhesion to the extracellular matrix and causes rearrangement of cytoskeletal structures [11]. In humans, in vivo experiments demonstrated that oxidative DNA damage is significantly more abundant in the TM cells of glaucoma patients. Additionally, both increased IOP and visual field damage were significantly related to the amount of oxidative DNA damage affecting TM cells [11,12]. The antioxidant status of biologic samples is regarded as an indicator of oxidative stress, and the measurement of total antioxidant status (TAS) is one of the most commonly used and useful procedures to test for prediction of oxidative status [13]. We previously investigated the presence of various mitochondrial abnormalities in various types of glaucoma and found an evidence of mitochondrial DNA (mtDNA) mutations and decreased mitochondrial respiratory activities [14,15]. Also, we found an association between certain mtDNA haplogroups and various types of glaucoma [16-18]. Additionally, we found an association between various glutathione S transferase (GST) gene polymorphisms (coded for a group of enzymes involved in detoxifying peroxidized lipids and various harmful toxins) and glaucoma [19]. Decreased GST function might interfere with the metabolism of oxidative intermediates and exacerbate the direct or indirect damaging effects of oxidative stress on the optic nerve. All of these studies by our group and others point to an oxidative stress mechanism that contributes to glaucoma pathogenesis [20].

We have recently shown that the “G” allele frequencies of both rs1048661 and rs3825942 SNPs of the lysyl oxidase-like 1 (*LOXL1*) gene differed between pseudoexfoliation glaucoma (PEG) patients and control subjects from Saudi Arabia (p=0.0056 and p<0.0001, respectively) [21]. These results suggest that this gene may play a role in the development of PEG, but do not answer many remaining questions about the etiology of PEG. These results also highlight the fact that other genetic causes or risk factors, either independently or in direct interaction with *LOXL1*, may play a role in the development of PEG.

Our aim is to evaluate total antioxidant levels in the plasma of PEG patients and compare this level with those of matching controls. Additionally, we will investigate the effect of the combined action of *LOXL1* mutation status and the TAS level on the development of PEG.

## Methods

### Study population

The study adheres to the tenets of the Declaration of Helsinki, and all participants signed an informed consent. The study was approved by the College of Medicine Ethical Committee (approval number # 08–657). Saudi Arab participants with clinically diagnosed PEG and healthy controls were recruited into the study at King Abdulaziz University Hospital (KAUH) in Riyadh, Saudi Arabia. All participants underwent a standardized detailed ophthalmic examination, which included measurement of intraocular pressure (IOP) by Goldmann applanation tonometry, slit lamp biomicroscopy, gonioscopy, and dilated examination of the lens and fundus. Subjects with PEG (n=54) were defined as those with clinical evidence of exfoliation material on the pupil margin or anterior lens surface, the presence of glaucomatous optic neuropathy with associated visual field loss in one or both eyes, and documented IOP ≥22 mmHg in either eye. Saudi Arab subjects (n=54) with no evidence of pseudoexfoliation material, normal anterior segments, healthy optic discs, and IOP <18 mmHg were recruited as controls.

### Plasma preparation and storage

Blood samples were collected in EDTA (EDTA) tubes. The tubes were centrifuged at 5,500× g for 5 min. The plasma layer was separated and stored at −80 °C until use and the buffy layer was used for DNA extraction.

### Plasma total antioxidant status

A widely used colorimetric-based assay available from Randox (Randox Laboratories Ltd., Crumlin, UK) was used to evaluate the plasma total antioxidant status. The assay involves brief incubation of ABTS^®^ (2,2’-Azinobis-di[3-ethylbenzthiazoline sulphonate]; Sigma-Aldrich Life Science, St. Louis, MO) with peroxidase (metmyoglobin) and hydrogen peroxide, resulting in the generation of ABTS^®+^ radical cations. The method detects a reduction in the generation of the ABTS^®+^ radical cations by plasma antioxidants, and the decrease in the generation of ABTS^®+^ radical cations is proportional to their total antioxidant concentration. The assay was performed in duplicate on an automated biochemical analyzer, ChemWell-T (Awareness Technology Inc., Palm City, FL), as per the manufacturer’s instructions (Randox Laboratories Ltd.). The analyzer was programmed using a ChemWell-T Assay Editor in the standard assay mode to add 200 µl of chromogen (metmyoglobin and ABTS^®^) and 4 µl of plasma sample/standard control/distilled water, incubate at 37 °C for 10 s, and read at 630 nm. This was followed by the addition of 40 µl of substrate (hydrogen peroxide in stabilized form), incubation at 37 °C for exactly 3 min, and measuring absorbance at 630 nm. A standard control (6-hydroxy-2,5,7,8-tetramethylchroman-2carboxylic acid) provided in the kit (Cat. No. NX 2332: lot specific concentration=2.08 mmol/l) was used for calibration. The total antioxidant status (TAS) was expressed as mmol/l.

### DNA Analysis

Five ml of peripheral blood was collected in EDTA tubes from all participating individuals. DNA was extracted using the illustra blood genomicPrep Mini Spin Kit from GE Healthcare (Buckinghamshire, UK), and stored at –20 °C in aliquots until required. PCR amplifications of the region encompassing both *LOXL1* SNPs (rs1048661 and rs3825942) were performed using the primers described previously [21], and presented in Table 1. Successfully amplified fragments were sequenced in both directions using M13 forward and reverse primers and the BigDye terminator v3.1 cycle sequencing kit (Applied Biosystems, Foster City, CA). Fragments were then run on the 3130*x*l Genetic Analyzer (Applied Biosystems) according to the manufacturer’s protocol. All of the sequenced fragments were then analyzed using SeqScape software v2.6 (Applied Biosystems). Allele frequencies for SNP rs1048661 and rs3825942 were confirmed by repeating the sequencing in both the forward and reverse directions.

**Table 1:** Primers used in the study.

**Exon**	**Primer Sequence**	**Annealing Temp. (°C)**	**Amplicon Size (bp)**
Promoter-F	**TGTAAAACGACGGCCAGT**AGGGACTGAGGGAGCACT	60	465
Promoter-R	**CAGGAAACAGCTATGACC**AGCCATGGTGACCCCTCT		
1A-F	**TGTAAAACGACGGCCAGT**TCCCAGCCTGTTGCTTATTC	60	789
1A-R	**CAGGAAACAGCTATGACC**GTTGCTGGGAGACGGAGGT		
1B-F	**TGTAAAACGACGGCCAGT**ATTCGGCTTTGGCCAGGT	60	810
1B-R	**CAGGAAACAGCTATGACC**CCGGAGCAGTTTCCAGTG		
1C-F•	**TGTAAAACGACGGCCAGT**GCTCAACTCGGGCTCAGA	57	553
1C-R•	**CAGGAAACAGCTATGACC**CGCCGTACTCCTCGTAGC		
2-F	**TGTAAAACGACGGCCAGT**TGCTCTCAATGTCATGCTCTT	60	209
2-R	**CAGGAAACAGCTATGACC**CGGGGACTATCCCAACACT		
3-F	**TGTAAAACGACGGCCAGT**GTGTCACTGTGCCCCAACC	60	232
3-R	**CAGGAAACAGCTATGACC**CCCAGAGGAGAAGTGGAAGA		
4-F	**TGTAAAACGACGGCCAGT**GAGAGGCCCAGGGAAGACTA	58	265
4-R	**CAGGAAACAGCTATGACC**CCTCCCCAACTCCTTATCCT		
5-F	**TGTAAAACGACGGCCAGT**GGGGTGGCTCTGGGAAAC	58	210
5-R	**CAGGAAACAGCTATGACC**GGGGGACATTGGACATGA		
6-F	**TGTAAAACGACGGCCAGT**CCCCTGACTAGACTCCCTTTC	60	234
6-R	**CAGGAAACAGCTATGACC**GTATCTCAGGTGGCCTTGC		
7-F	**TGTAAAACGACGGCCAGT**CTACTTTGCAGCCCCTCATT	60	410
7-R	**CAGGAAACAGCTATGACC**CCAGGCCCAAACTAGCTG		

**F**: Forward; **R**: Reverse; ^•^SNPs rs1048661 and rs3825942 were amplified with this primers set.  Bold and underlined sequences are those of the M13.

### Statistical Analysis

Statistical analysis was performed with SPSS version 19.0 (IBM Corp., Armonk, NY), MedCalc version 11.6 (MedCalc Software, Mariakerke, Belgium) and Statpac version 11 (StatPac Inc., Bloomington, MN). Descriptive analysis was conducted to describe and understand the sample. Student’s *t*-test was used to compare the mean TAS across groups, while the Mann–Whitney U test was used to compare the mean TAS within groups (between different genotypes). Binary logistic regression analysis was performed to estimate the impact and direction of the effect of each risk factor on patients with pseudoexfoliation glaucoma. A p-value of <0.05 was considered of statistical significance, while all tests were supported by the corresponding 95% confidence intervals.

## Results

A total of 108 individuals were recruited for this study with the mean (±SD) age of 67.7 (10.5) years, ranging from (32–85). The majority of the samples were males: 74 (68.5%), compared to females: 34 (31.5%). Out of the 108 cohort, 54 cases were confirmedly diagnosed as PEG cases, while another 54 matching ethnicity, age, and sex, but free from glaucoma by examination, served as controls (see Methods for inclusion and exclusion criteria). The gender constitution of both patients and controls was exactly matching: 37 (68.5%) males and 17 (31.5%) females. Meanwhile, the mean (±SD) age of both cases and controls were: 67.6 (10.4), range 33–83, and 67.7 (10.6), range 32–85, respectively, which did not statistically differ (p=0.942). However, the mean total antioxidant (TAS) value was lower among patients: 0.87 (0.241), range 0.9–1.41 than controls: 1.07 (0.234), range 0.72–1.94, where this difference was statistically significant (p<0.0001: 95% CI: −0.295–0.114; Table 2).

**Table 2:** Demographic characteristics and TAS level of both cases and controls

**Number**	**n=54**	**n=54**	**p-value**
M/F	37/17	37/17	
Age (years)	67.72 (10.60)	67.57 (10.43)	0.9418
TAS (mmol/l)	1.07 (0.234)	0.866 (0.241)	<0.0001

Values are expressed as mean±standard deviation (SD). P-values are determined by unpaired Student’s *t*-test. M – male: F – female: TAS – total antioxidant levels.

Disaggregating these data by gender yielded no apparent difference between males and females either among cases or controls. This suggests that gender probably does not play a role in the average antioxidant value (Table 3).

**Table 3:** Mean TAS value by gender among cases and controls.

**Gender**	**Patients**	**Controls**
**Male**		
Mean (SD)	0.849 (0.26)	1.1 (0.25)
Range [min–max]	[0.09–1.41]	[0.72–1.94]
**Female**		
Mean (SD)	0.9 (0.20)	0.99 (0.18)
Range [min–max]	[0.37–1.18]	[0.78–1.4]

As for SNP rs1048661 (g.5758 G>T) among patients, 43 (79.6%) individuals were of genotype G/G, while 11 (20.4%) were of genotype G/T. As for the controls, 31 (57.4%) individuals had the genotype G/G, 21 (38.9%) had the G/T genotype, while two cases (3.7%) had the T/T genotype. Within each group, the mean TAS value also varied across different categories, however insignificantly (p-values within patients and controls were: 0.373 and 0.808, respectively).

Within cases, 53 (98.1%) of patients had the G/G genotype, while only one case (1.9%) had G/A. Alternatively, among controls, 46 (85.2%) had G/G and eight (14.8%) had G/A. Although it was not possible to compare mean values of TAS among cases with different genotypes, comparing mean TAS among different categories of controls showed that the apparently slight difference is not statistically significant (p=0.922).

Consequently, comparing cases with the G/G genotype to controls with the G/G genotype, there was a highly significant difference between the two groups in terms of the mean TAS value: 0.049 (p<0.0001). However, when we compared cases with G/T genotype to controls with the same genotype, there was no difference (p=0.131; Table 4).

**Table 4:** Mean TAS value by rs1048661 category among cases and controls.

**Genotype**	**Case**	**Control**	**p value**
**G/G**			
Mean (SD)	0.857 (0.24)	1.089 (0.27)	<0.0001
Range [min–max]	[0.09–1.41]	[0.72–1.94]	
**G/T**			
Mean (SD)	0.897 (0.26)	1.054 (0.178)	0.131
Range [min–max]	[0.37–1.18]	[0.76–1.45]	
**T/T**			
Mean (SD)	NA	0.95 (0.071)	-
Range [min–max]		[0.9–1]	

P-values are determined by Mann–Whitney U test.

As for SNP rs3825942 (g.5758 G>A) among patients, 53 (98.1%) individuals were of genotype G/G, while only one case (1.9%) had G/A. Among controls, 46 (85.2%) had G/G and eight (14.8%) had G/A. Although it was not possible to compare mean values of TAS among cases with different mutations, comparing mean TAS among different categories of controls showed that the apparently slight difference is not statistically significant (p=0.922). Alternatively, comparing patients with G/G to controls with G/G, there was a highly significant difference between the two groups in terms of the mean TAS value (p<0.0001; Table 5).

**Table 5:** Mean TAS value by rs3825942 category among cases and controls.

**Genotype**	**Case**	**Control**	**p-value**
**G/G**			
Mean (SD)	0.864 (0.24)	1.075 (0.25)	<0.0001
Range [min-max]	[0.09–1.41]	[0.72–1.94]	
**G/A**			
Mean (SD)	NA	1.039 (0.10)	-
Range [min-max]		[0.89–1.18]	

P-values are determined by Mann–Whitney U test.

With the aim of evaluating the impact of age, sex, and the mutation, in addition to the mean TAS value in patients with pseudoexfoliation, a logistic regression analysis was conducted using diseased/not diseased as the outcome of interest (the dependent variable). Results show that, controlling for all other variables, mean TAS value (p<0.0001) and the mutation G/G in rs3825942 (p=0.041) are significant risk factors for PEG.

## Discussion

We obtained plasma samples from 54 extensively diagnosed PEG patients and 54 controls. Our control group was carefully selected in terms of age, sex, and ethnicity. We established that there were free of glaucoma by extensive clinical examination. Selecting the control group with age and sex matching to the patient group was of particular importance, as those factors can influence the TAS level, and thus can affect the results independent of the glaucoma status [22]. The results obtained here demonstrated a marked decrease in TAS in PEG patients compared to controls. The literature shows inconsistent findings regarding antioxidant activity in serum and aqueous humor in glaucoma patients. Yildrim and colleagues [23] studied 40 patients with glaucoma and found no association between glaucoma and systemic myeloperoxidase or catalase enzyme activity. Yuki and colleagues [24] found an increase in the serum total antioxidant status of patients with normal-tension glaucoma compared to matching controls. In contrast, Sorkhabi et al. [25] showed that the serum level of TAS in patients with primary open angle glaucoma was lower than that of cataract controls. Gherghel and colleagues [26] concluded that glaucoma patients exhibit low levels of circulating glutathione, suggesting compromised oxidative defense. The only study of total reactive antioxidant potential (TRAP) and antioxidant enzymes in aqueous humor was performed by Ferreira and colleagues, and showed significantly decreased TRAP values and increased superoxide dismutase and glutathione peroxidase activity in glaucoma patients [27]. An antioxidant is by definition any substance that, when present at low concentration compared with those of an oxidizable substrate, significantly delays or prevents oxidation of that substrate. The antioxidant defense system comprises a variety of molecules: enzymes such as superoxide dismutase, catalase, or glutathione peroxidase that are capable of catalytically removing free radicals and other reactive species; proteins, such as transferrins or haptoglobins, that minimize the availability of pro-oxidants such as iron or copper ions; heat shock proteins that protect biomolecules against damage; and low-molecular mass molecules such as α-tocopherol, ascorbic acid, or glutathione capable of scavenging ROS and RNS. The composition of antioxidant defenses differs from tissue to tissue and from cell type to cell type [28]. All of these compounds and more exist in human plasma. Antioxidants that can be found in human plasma vary, and can be summarized mainly in the following compounds: albumin, ceruloplasmin, ferritin, ascorbic acid, α-tocopherol, β-carotene, lycopene, reduced glutathione, bilirubin, glutathione peroxidase, uric acid, catalase, and superoxide dismutase [29]. The exact mechanism of how oxidative stress contributes to glaucoma pathogenesis remains speculative. Glaucomatous optic neuropathy implies loss of retinal ganglion cells, including their axons, and a major tissue remodeling, especially in the optic nerve head. Although increased intraocular pressure is a major risk factor for glaucomatous optic neuropathy, there is little doubt that other factors such as ocular blood flow play a role as well [30]. Mechanisms leading to glaucomatous optic neuropathy are not yet clearly understood. There is, however, increasing evidence that both an activation of glial cells and oxidative stress in the axons may play an important role [31]. Glial cells may be activated by mechanical stress via activation of the epidermal growth-factor receptor, or by ischemic stress via an increase in endothelin. Several factors can systemically or locally increase oxidative stress. In glaucoma, an unstable ocular blood flow leading to repeated mild reperfusion seems to be most relevant in inducing oxidative stress. The simultaneous production of nitric oxide in the astrocytes and of superoxide in the mitochondria of the axons leads to the production of the damaging peroxynitrite [32]. Therapeutically, we need to reduce intraocular pressure, stabilize ocular blood flow, and reduce oxidative stress. The fact that the mean total antioxidants were reduced in PEG patients compared to age-, sex-, and ethnicity-matched healthy controls certainly contributes to the creation of an oxidative stress status and, in such a mechanism as described above, such a situation may contribute to glaucoma pathogenesis.

Our total antioxidants method employed here measured the total antioxidant status and not a particular compound or byproduct. A detailed examination of those individual antioxidants separately might help to identify a particular antioxidant that is severely decreased and thus provide a new therapeutic agent for glaucoma. We have to acknowledge that the systemic decrease in antioxidants might not reflect the exact situation at the anterior segment structures, which are exposed to free radicals and thus more directly involved in the formation and development of glaucoma through the oxidative stress mechanism.

We recently shown that the “G” allele frequencies of both rs1048661 and rs3825942 SNPs of *LOXL1* differed between pseudoexofoliation glaucoma (PEG) patients and control subjects from Saudi Arabia (p=0.0056 and p<0.0001, respectively) [21]. These results were in agreement with previous similar studies in various populations. When we compared cases with the G/G genotypes (for both *LOXL1* SNPs) to controls with the same genotype, it was clear that there was a highly significant difference between the two groups in terms of the mean TAS value. To the best of our knowledge this is the first time the combined effect of the TAS and the *LOXL1* PEG-risk SNPs was investigated. The mechanism by which these SNPs may play a role in the development of PEG is quite different from that of the oxidative stress mechanism suggested here as a result of decreased TAS. LOXL1 belongs to a group of proteins responsible for catalyzing the oxidative deamination of lysine residues of tropoelastin (summarized by Hewitt et al. [33]). In turn, this deamination causes spontaneous cross-linking and formation of elastin polymer fibers. Thus, mutations in *LOXL1* are expected to affect elastin formation. Loss of elastin in turn causes iridolenticular friction, which leads to loss of exfoliation material (XFM) from the anterior lens surface and disruption of the iris pigment epithelium, resulting in pigment deposition in the trabecular meshwork, which also produces XFM locally. The primary cause of chronic pressure elevation appears to be the active involvement of trabecular cells. Thus it is clearly a different mechanism from the proposed oxidative stress indicated by the decreased level of total antioxidants reported here.

In summary, our findings report a TAS decrease in the plasma of PEG patients and suggest that TAS may have an important role in the pathogenesis of glaucoma. The combined effect of the “G” allele and the decreased TAS clearly contribute to the development of PEG, but through different mechanisms, one mechanism through *LOXL1* and the resultant iridolenticular friction, and the other through oxidative stress marked by decreased TAS.
